# Bisphenol A shapes children’s brain and behavior: towards an integrated neurotoxicity assessment including human data

**DOI:** 10.1186/s12940-020-00620-y

**Published:** 2020-06-09

**Authors:** Vicente Mustieles, Mariana F. Fernández

**Affiliations:** 1 University of Granada, Center for Biomedical Research (CIBM), Granada, Spain; 2 Consortium for Biomedical Research in Epidemiology & Public Health (CIBERESP), Madrid, Spain; 3 Instituto de Investigación Biosanitaria (ibs. GRANADA), Granada, Spain; 4 Department of Radiology and Physical Medicine, School of Medicine, University of Granada, Granada, Spain

**Keywords:** Bisphenol, BPA, Endocrine disruptor, Behavior, Neurodevelopment, Brain, Risk assessment, Health policy, CLARITY-BPA, HBM4EU

## Abstract

Concerns about the effects of bisphenol A (BPA) on human brain and behavior are not novel; however, Grohs and colleagues have contributed groundbreaking data on this topic in a recent issue of *Environmental Health*. For the first time, associations were reported between prenatal BPA exposure and differences in children’s brain microstructure, which appeared to mediate the association between this exposure and children’s behavioral symptoms. Findings in numerous previous mother-child cohorts have pointed in a similar worrying direction, linking higher BPA exposure during pregnancy to more behavioral problems throughout childhood as assessed by neuropsychological questionnaires. Notwithstanding, this body of work has not been adequately considered in risk assessment. From a toxicological perspective, results are now available from the CLARITY-BPA consortium, designed to reconcile academic and regulatory toxicology findings. In fact, the brain has consistently emerged as one of the most sensitive organs disrupted by BPA, even at doses below those considered safe by regulatory agencies such as the European Food Safety Authority (EFSA). In this Commentary, we contextualize the results of Grohs et al. within the setting of previous epidemiologic and CLARITY-BPA data and express our disquiet about the “all-or-nothing” criterion adopted to select human data in a recent EFSA report on the appraisal methodology for their upcoming BPA risk assessment. We discuss the most relevant human studies, identify emerging patterns, and highlight the need for adequate assessment and interpretation of the increasing epidemiologic literature in this field in order to support decision-making. With the aim of avoiding a myopic or biased selection of a few studies in traditional risk assessment procedures, we propose a future reevaluation of BPA focused on neurotoxicity and based on a systematic and comprehensive integration of available mechanistic, animal, and human data. Taken together, the experimental and epidemiologic evidence converge in the same direction: BPA is a probable developmental neurotoxicant at low doses. Accordingly, the precautionary principle should be followed, progressively implementing stringent preventive policies worldwide, including the banning of BPA in food contact materials and thermal receipts, with a focus on the utilization of safer substitutes.

## A key contribution to a hot topic

In a recent issue of *Environmental Health*, Grohs and colleagues [1] examined whether prenatal BPA exposure was associated with offspring’s brain microstructure and behavior in a sub-cohort of 98 mother-child pairs from the Canadian Alberta Pregnancy Outcomes and Nutrition (APrON) study. Healthy children, free of any neurodevelopmental disorder, underwent a diffusion magnetic resonance imaging (MRI) scan at 2–5 years of age to assess white matter microstructure in major brain regions. Seventy-seven out of the 98 initial children also had a postnatal urine sample collected at 3–4 years, and 56 had the Child Behavior Checklist (CBCL) test completed by their parents within 6 months of the scan.

For the first time, Grohs and colleagues found that higher maternal urinary BPA concentrations, at around 17 weeks of gestation, were associated with a less developed white matter in the splenium (subregion of corpus callosum) and in the right inferior longitudinal fasciculus (tract connecting the anterior temporal and occipital lobes) [1]. Moreover, white matter differences in the splenium mediated the association between prenatal BPA exposure and children’s internalizing problems (e.g. anxiety and depression symptoms). Postnatal urinary BPA concentrations were not associated with either brain microstructure or behavior [1].

The greatest strength and added value of the study by Grohs et al. was the implementation of a quantitative effect biomarker (MRI) to assess brain structure, which indeed provided a neural correlate for altered behavioral function in response to prenatal BPA exposure, thus increasing the internal validity of the findings. Collectively, these results suggest that prenatal -rather than postnatal- BPA exposure may shape and “organize” offspring’s brain structure leading to lasting adverse effects on children’s behavior. Among the main limitations, the study analyzed a small sample size and characterized BPA exposure using only one spot urine sample.

If this was the first epidemiologic study to report a potential effect of prenatal BPA exposure on children’s behavior, we should be cautious interpreting these results. However, this is far from reality: many previous mother-child cohorts, with small, medium and large sample sizes have reported quite consistent associations between prenatal BPA exposure and a higher risk of subclinical behavioral problems throughout childhood, as reviewed by Mustieles et al. and Ejaredar et al. [2, 3]. Importantly, the quantifiable measure of children’s brain structure implemented by Grohs and colleagues provides support to previous associations observed using validated neuropsychological questionnaires.

## Emerging patterns in a growing literature

Numerous mother-child cohorts have studied the relationship between prenatal urinary BPA concentrations and children’s behavior (Table 1) [2, 3]. Overall, most of them have reported associations between higher prenatal BPA exposure and at least one behavioral domain (internalizing, externalizing or both) [2, 3]. Although reported associations with internalizing and externalizing behaviors is usually considered as an inconsistency across studies, this aspect is in line with toxicological studies in which both types of behaviors have been reported in response to BPA exposure [2, 4]. Only a few prenatal studies (3 out of 13 cohorts) did not report any association, probably due to their small sample size and/or age at behavior assessment (Table 1).

**Table 1:** Summary of main results reported in mother-child cohorts addressing the relationship between prenatal urinary BPA concentrations and children’s behavior

Cohort study	Sample size	Prenatal urine(s) samples	Association*	Internalizing or externalizing symptoms	Boys, girls or both
**HOME (USA)** Braun et al. 2009	249	3 (gestation weeks 16th and 26th, and delivery)	Yes	Externalizing at 2 yrs.	Girls
Braun et al., 2011	244		Yes	Internalizing at 3 yrs.	
Braun et al., 2014	175	2 (gestation weeks 16th and 26th)	No	Autistic behaviors at 4–5 yrs.	n/a
Braun et al., 2017	178	2 (gestation weeks 16th and 26th)	Yes	Externalizing behavior trajectory throughout ages 2, 3, 4, 5 and 8	Girls
**MSCEHS (USA)** Miodovnik et al., 2011	137	1 (gestation week 31th)	No (Yes, when 6 outliers were removed from the analysis)	At 7–9 yrs.	n/a
**CCCEH (USA)** Perera et al. 2012	198	1 (gestation week 34th)	Yes	Both at 3–5 yrs.	Boys
Roen et al., 2015	250		Yes	Both at 7–9 yrs.	
Perera et al., 2016	241		Yes	Internalizing at 10–12 yrs.	
**CHAMACOS (USA)** Harley et al. 2013	292	2 (gestation weeks 14th and 26th)	Yes	Internalizing, and aggressive behavior at 7 yrs	Boys
**SFFII (USA)** Evans et al., 2014	153	1 (gestation week 27th)	Yes	Both at 5 yrs.	Boys
**INMA-Sabadell (Spain)** Casas et al., 2015	438	2 (1st and 3rd trimester)	Yes	Externalizing at 7 yrs.	Boys
**EDEN (France)** Philippat et al., 2017	529	1 (2nd trimester)	Yes	Internalizing at 3 yrs. Externalizing at 5 yrs.	Only boys were recruited
**MIREC (Canada)** Braun et al., 2017	812	1 (gestation week 12th)	Yes	Internalizing at 3 yrs.	Boys
**EDC (Korea)** Lim et al., 2017	304	1 (gestation week 20th)	Yes	Externalizing (social problems) at 4 yrs.	Girls
**OCC (Denmark)** Jensen et al., 2019	658	1 (gestation week 28th)	No	At 2–4 yrs.	n/a
**CHECK (Korea)** Kim et al., 2018	140	1 (delivery)	No	At 1–2 yrs.	n/a
**APrON (Canada)** Grohs et al., 2019	98	1 (gestation week 17th)	Yes	Internalizing at 4–5 yrs.	Both
**S-MBCS (China)** Li et al., 2020	745	1 (gestation weeks 12th–16th)	Yes	Both at 2–4 yrs.	Boys

For conciseness and due to space limitations, all references included in this table can be consulted in the Supplemental Table 1 of the Supplementary Appendix. *A *p*-value ≤ 0.05 defined the existence of an association. However, other parameters such as the internal validity of associations were also considered. *n/a* Not applicable, since no association was reported. *APrON* Alberta Pregnancy Outcomes and Nutrition, *CHAMACOS* Center for the Health Assessment of Mothers and Children of Salinas, *CHECK* Children’s Health and Environmental Chemicals in Korea, *CCCEH* Columbia Center for Children’s Environmental Health, *EDC* Environment and Development of Children, *EDEN* Study on the Pre- and Early Postnatal Determinants of Child Health and Development, *HOME* Health Outcomes and Measures of the Environment Study, *INMA* Environment and Childhood Project, *MIREC* Maternal-Infant Research on Environmental Chemicals, *MSCEHS* Mount Sinai Children’s Environmental Health Study, *OCC* Odense Child Cohort, *S-MBS* Shanghai-Minhang Birth Cohort Study, *SFFII* Study for Future Families II

The findings by Grohs at al. reinforce that the prenatal period is the most critical window of BPA exposure for behavior, in line with previous studies (Supplemental Table 2), suggesting an organizational effect in the fetal brain [1]. Although prenatal BPA exposure was significantly associated with increased internalizing problems in the APrON cohort, an imprecise but potential association towards externalizing problems was also apparent and cannot be ruled out based on the small sample size of the population studied [1].

Most of the previous prenatal studies also reported sex-specific associations (Table 1). This in line with rodent studies, that have shown effects in females, males or both depending on the behavioral outcome studied and/or timing of BPA exposure [4]. In humans, prenatal associations were mainly -but not uniquely- reported among boys (Table 1). Grohs et al. however, did not find sex-specific associations with either children’s brain structure or behavior [1]. Nonetheless, this possibility cannot be ruled out based on the small sample size analyzed, likely underpowered compared to previous mother-child cohorts. Additional observational studies are needed to further confirm patterns of sex-specific associations.

Cognitive and other behavioral domains including externalizing, internalizing, emotional and social problems have been assessed in relation to prenatal and/or postnatal BPA exposure (Supplemental Table 3). While frequent associations have been reported between pre- and postnatal exposure and externalizing/internalizing behaviors, relations with cognitive outcomes have been largely null, with the potential exception of poorer working memory in a few studies, as discussed in detail in Rodríguez-Carrillo et al. [5] (Supplemental Table 3). Interestingly, the difference between cognitive and other behavioral outcomes cannot be explained by chance alone, and could be in line with the known ability of BPA to alter the expression of estrogenic nuclear receptors in the brain, leading to a predominant impact on sexually-dimorphic brain areas and behaviors [5]. Regarding cognition, we are not aware of previously published results for BPA in the same population studied by Grohs and coworkers [1].

Based on the commented epidemiologic evidence (Table 1 and Supplementary Appendix), five emerging patterns can be proposed: i) Most studies have reported associations between prenatal BPA exposure and children’s behavioral problems; ii) Prenatal BPA exposure is more consistently associated with children’s behavior than postnatal exposure; iii) BPA exposure is more frequently associated with externalizing/internalizing problems than cognitive outcomes; iv) Both internalizing and externalizing symptoms seem to coexist; and v) Associations usually, but not always, differ by sex.

With the exception of the HOME, CHAMACOS and INMA-Sabadell birth cohorts, which assessed prenatal urinary BPA concentrations at two-three time points during gestation, the remaining studies collected one spot urine sample, in either the second or third trimester to characterize BPA exposure (Table 1). Grohs et al. also used one urine sample [1]. Since BPA is rapidly metabolized and excreted in urine, and exposure patterns are episodic, using one spot urine sample may lead to exposure misclassification. This means that when all studies in a given field are taken together, there is a greater tendency to obtain null results, the so-called attenuation bias [6]. In other words, most of the associations observed with behavior would be systematically underestimated. Despite this limitation, the increasing consistency of patterns observed across different human populations probably reflects the tip of the iceberg. This important aspect should be carefully considered when interpreting the epidemiologic literature.

## Biological plausibility

That prenatal BPA exposure impairs offspring’s brain and behavior in experimental animals through different mechanisms is a fact difficult to neglect based on dozens of independent academic peer-reviewed studies [4]. Surprisingly, most of these studies have not been usually considered by risk assessors, based on issues related to study design, animal strains, animal number per dose group, and/or the endpoints investigated (i.e. molecular and functional changes vs. gross endpoints such as organ weights) [7]. To reconcile these divergent views, the Consortium Linking Academic and Regulatory Insights on BPA Toxicity (CLARITY-BPA), was developed by three US agencies (National Institute of Environmental Health Sciences – NIEHS; National Toxicology Program – NTP; and US Food and Drug Administration – FDA). Briefly, CLARITY-BPA conducted a traditional regulatory-style toxicology study in an FDA facility, known as the “core” study, in conjunction with academic laboratories to test previous hypothesis following the same protocol, and sharing the same animals and/or tissues [7]. CLARITY-BPA evaluated a wide range of BPA doses (2.5, 25, 250, 2500, or 25,000 μg/kg per day). Although this dose range is several orders of magnitude higher than the human exposure range, the two lowest BPA doses tested are considered of relevance to humans.

In the CLARITY-BPA program, the developing brain has again emerged as probably the most sensitive organ disrupted by BPA, as reviewed in detail by Patisaul [7]. The most consistent finding between previous experimental studies and CLARITY-BPA data is that prenatal exposure to the lowest BPA dose tested (2.5 μg/kg per day) was able to alter the gene expression of estrogen receptors (ERs) in multiple brain regions [7], in line with alterations in the volume of the anteroventral periventricular (AVPV) nucleus of the hypothalamus, implicated in sexually dimorphic physiology and behaviors [7]. Untargeted transcriptomics additionally identified the highest number of differentially expressed genes in the male hypothalamus and female amygdala [7]. However, in contrast to previous findings showing effects of developmental BPA exposure on anxiety and exploratory behaviors, CLARITY-BPA found subtle and sporadic behavioral modifications [7]. Importantly, expected normal behavioral differences between male and female animals in the unexposed controls were not detected, suggesting some specificities of the Sprague-Dawley (SD) rat strain used, which appeared to be quite insensitive to test BPA-related sex-specific behavioral outcomes previously observed in other SD strains [7].

Overall, CLARITY-BPA supports that in utero BPA exposure could predispose later responses in sexually dimorphic brain areas to estradiol and other hormones throughout development, providing a mode of action for BPA effects on brain and behavior. Results from this consortium also highlights that the expected endpoints are subtle modifications in neuroendocrine function and behavior, rather than gross alterations in brain weight among other overt damages [7, 8]. However, these subtle modifications are not usually evaluated in regulatory studies, and therefore should be systematically collected from the available peer-reviewed academic studies.

Some of the lessons derived from the CLARITY-BPA program include: i) The existence of low-dose BPA effects that were not predicted by higher doses; ii) Unequivocal alterations of ER expression across the rat brain; and iii) Multiple low-dose adverse effects, including the mammary gland, prostate, kidney and body weight among others [7, 8].

Among the concerns raised by CLARITY-BPA are the animal strain used, and probably the particular lineage of that strain, which seems to influence the specific effects observed in response to BPA. Apart from the abovementioned lack of normal sexually-dimorphic behaviors in the experimental animals, the strain used in CLARITY-BPA was also quite insensitive to low doses of known estrogens such as ethynyl estradiol (EE) [8]. These limitations could be counteracted by adopting an integrative and systematic approach to gather available animal data from several strains, considering the whole academic literature between BPA exposure and neurotoxicity.

Although animal research provides invaluable information on modes of action and potential adverse effects, there are inherent limitations when extrapolating results from experimental animal models to humans. The difference is maximized in the case of brain structure and function. For example, testosterone is aromatized to estradiol, masculinizing the rodent brain acting through ERs [7, 9]. On the contrary, the sexual differentiation of the primate and human brain appears to be primarily driven by testosterone acting without an estrogen intermediary [10, 11]. Consequently, the sexually dimorphic brain and behavioral effects frequently reported for BPA in both rodents [4] and humans [2] could be compared to some extent, but perhaps not in the same direction nor occurring through the same mechanisms of action. Based on the previous limitations, the translatability of CLARITY-BPA and animal models to humans has been questioned [12]. Thus, Hagobian (2019) argues that to truly understand its health risks, BPA should be administered to humans under controlled conditions [12]. Although a preliminary intervention trial dosing adult human volunteers with BPA has shown short-term alterations in glucose and insulin parameters [12], this approach appears unacceptable in pregnant women due to the potential risk of teratogenic and long-lasting effects on the offspring. Therefore, the valuable information available for BPA from mother-child birth cohort studies should be carefully considered in decision-making.

## Human evidence in risk assessment

To understand the available human data, there are several contextual characteristics that must be considered when interpreting the relationship between BPA exposure and children’s neurobehavior (Table 2). More than 90% of Europeans and Americans have detectable concentrations of BPA in their urine, and diet is considered the main -but not unique- contributor to exposure levels [2]. The expected consequences of human BPA exposure would be subtle effects at the subclinical level, in line with results at low-doses from the CLARITY-BPA program. However, given that BPA exposure is universal and chronic, even if subtle effects were occurring, they could affect the whole human population, with a special emphasis on the developing fetus.

**Table 2:** Key characteristics of the BPA-neurobehavior context, and their implications for interpreting the available human evidence

BPA	Neurobehavior	Implications
Ubiquity	Exquisite sensitivity of the developing brain	The whole population may be at risk, with a special consideration for the developing fetus
Low-dose effects cannot always be predicted from high doses	Non-traditional endpoints such as behavior and molecular brain changes are more realistic	Subtle subclinical effects at a population level are expected, rather than obvious clinical effects at the individual level
Multiple modes of action	Windows of particular susceptibility	BPA exposure at different points in gestation may be linked to slightly different neurobehavioral endpoints
Challenging human exposure characterization	Indirect behavior assessment with questionnaires completed by parents or teachers	The expected bias arising from limitations when evaluating both BPA exposure and behavioral outcomes across studies is a tendency to underestimate potential effects
Exposure to complex chemical mixtures	Organizational vs. activational effects in the brain	Human BPA exposure always coexist and probably interacts with many other chemicals. Prenatal exposures may “organize” brain areas, leading to long-lasting effects in the offspring

The multiple mechanisms of action on the brain described for BPA mean that different brain areas and behavioral patterns could be affected depending on the particular window of brain development and susceptibility [4]. In relation to Grohs et al. findings, the development of the human splenium is thought to emerge around the weeks 18–19 of gestation [13], just the critical window in which the authors assessed prenatal BPA exposure [1]. Exposure during other periods could be linked to changes in other brain areas and/or behavioral domains.

There is consensus that twenty-first century chemical risk assessments need to integrate multiple streams of evidence (mechanistic, animal, and epidemiology data) [14], and systematic frameworks to achieve this objective have been proposed [15]. However, risk assessors seem to still remain reluctant to integrate human data, based on the limitations of observational studies. Thus, the recent report of the European Food and Safety Authority (EFSA) on the study appraisal methodology for the forthcoming BPA risk assessment, has included an “all-or-nothing” judgment for the selection of observational studies: [*“only studies using a minimum of three time 24-hour urine samples could be considered as reliable (+ or ++)”*] [16]. Following this criterion, it is currently not possible to find any human study in the PubMed database. This means that, out of hundreds of epidemiologic studies available for BPA, none will be considered during the next risk assessment. Is there a scientific rationale to support this highly exclusive exposure criterion? Unfortunately, we could not find any strong justification behind this decision throughout the report, nor any reference in the bibliographic list focused on the exposure characterization of BPA [16]. This vague but decisive requisite appears in the best of the cases thoughtless, and biased in the worst.

The strict exclusion criterion followed in the EFSA report [16] seems to be based on findings from Sun et al. [17]. Although the authors estimated that three 24-h urine samples collected throughout a year could provide a theoretical intraclass correlation coefficient (ICC) near to 0.8 (i.e. attenuation bias would be limited to 20%), they reported an empirical ICC of 0.39 for BPA using two 24-h urine samples [17]. This empirical ICC is considered low to moderate, and not optimal to substantiate the strict criterion proposed by the EFSA report [16], even more when the collection of 24-h urine samples in population studies is cumbersome and very rarely performed. Moreover, the unique study that has so far evaluated BPA using four 24-h urine samples collected over 1 year, reported an ICC of 0.57 [18]. On the contrary, recent empirical research shows that the most important aspect is to count with a higher number of urine samples collected during the expected critical window, but not necessarily 24-h samples [6]. Thus, Vernet and colleagues have demonstrated the feasibility of collecting multiple repeated spot urine samples during gestation, and pooling them to provide a cost-effective measurement, achieving an ICC higher than 0.8 for BPA [6].

Human BPA exposure characterization is challenging due to the episodic nature and rapid metabolization of this compound. We agree that exposure characterization must be improved as much as possible for short-lived chemicals with high within-individual variability such as BPA [6], and that more weight must be given to studies using several urine samples compared to those only using one. Our disagreement with the EFSA report [16] lies on an imprudent dismissal of all the human BPA evidence based on non-justified criteria. Indeed, one of Bradford Hill’s criteria for assessing causality in observational studies is some consistency and coherence across different populations. Thus, for a specific outcome such as children’s behavior, even if one study would have assessed prenatal BPA exposure using three 24-h urine samples throughout pregnancy, it would not be as informative as 10 studies evaluating prenatal BPA exposure using only one spot urine sample.

The trend in future cohort studies, the so-called “third generation cohorts” such as the recent SEPAGES cohort in France [19], will be to provide a greatly improved exposure characterization to non-persistent chemicals through repeated sampling and subsequent pooling of spot urine samples in order to counteract the expected attenuation bias, while maintaining cost-effectiveness. Ironically, a hypothetical future “third generation cohort” study reporting associations between prenatal exposure to BPA and child behavior problems using this improved exposure characterization strategy would also be excluded from the forthcoming EFSA assessment [16].

Regarding the last characteristic of the BPA-neurobehavior context, human BPA exposure never occurs in isolation, but instead in complex low-dose chemical mixtures, with often unpredictable consequences. Arguably, co-exposure to dozens of environmental chemicals with described neurotoxic potential is expected to converge and sum up to BPA’s adverse effects on the brain [20]. A special consideration for real-world mixtures, perhaps through an additional safety factor may be proposed in future risk assessments.

## Novel biomarkers of brain structure/function vs. neurobehavioral tests

There is no perfect tool for assessing the effects of endocrine disruptors on neurodevelopment. Validated questionnaires, such as the CBCL, are widely used to assess children’s behavior in epidemiologic studies for practical reasons, providing very useful information [2]. However, these tests are usually completed by parents and/or teachers, who are not free from a subjective perception of their child’s behavior, and although validated, have been considered as a limitation in previous EFSA risk assessments [*“imprecise/unreliable outcome (neurobehavioral parameters scored using parent-reported but validated methods)”*, Appendix D.1.3] [16]. Despite potential limitations, questionnaire data should be given full consideration in risk assessment. A repeated longitudinal assessment of child behavior trajectory is preferred and feasible in mother-child cohorts [21]. This is important since in utero BPA exposure is expected to cause “organizational” effects in the brain, as supported by animal studies as well as Grohs et al. findings [1], potentially explaining long-lasting effects on children’s behavior [21].

Apart from neuropsychological tests, the implementation of complementary measurements such as MRI scan by Grohs et al. offers an objective and low-invasive measure of brain microstructure that complements and enriches previous data [1]. Of outermost importance, differences in particular brain tracts mediated BPA associations with children’s behavior, providing a neural correlate that supports functional changes in behavior and increases the confidence in these findings [1]. Established mother-child birth cohorts in which MRI scans have been already performed in hundreds of children may constitute a timely and cost-effective opportunity to confirm the path started by Grohs and colleagues.

The investigation of potential intermediate molecular biomarkers of brain function, which may biologically support associations with child behavior, constitutes a complementary area of emerging interest. For example, brain-derived neurotrophic factor (BDNF) constitutes a promising effect biomarker candidate. In utero BPA exposure has been shown to induce DNA methylation changes in the transcriptionally relevant region of the BDNF gene in the hippocampus of mice, which correlated with its methylation status in blood [22]. Moreover, consistent BDNF DNA methylation changes in the cord blood of children prenatally exposed to higher maternal BPA concentrations were also shown [22]. The implementation of novel biomarkers of brain function is being studied under the umbrella of the Human Biomonitoring for Europe (HBM4EU) initiative, in which BDNF, as well as other related biomarkers, will be assessed in different European populations, and at different levels of biological organization (DNA, RNA, circulating protein levels, etc.) [23]. At the same time, adverse outcomes pathway (AOP) networks supporting the implementation of these biomarkers are being developed [23]. As an advantage compared to brain structure measurements, molecular biomarkers can be assessed at a large scale and in accessible biological samples such as blood.

## Conclusions

All fields are subjected to inherent limitations. Cell-based models explore mechanisms of action, normally at high doses that cannot be easily extrapolated to humans, neither account for complex homeostasis. Animal models provide information on the whole organism, but translatability is not warranted. At this stage, it is impossible to ignore the growing epidemiological evidence linking prenatal BPA exposure with children’s behavior. However, this literature has not been adequately considered in risk assessment. Lack of data is no longer a problem. How the available data is compiled, selected and interpreted is the key issue explaining divergent evaluations. To avoid a myopic or biased selection of a few studies in traditional risk assessment procedures, we propose a future BPA reevaluation focused on neurotoxicity and based on a systematic and comprehensive integration of available mechanistic, animal, and human data [15]. Even in the face of uncertainties, we especially highlight the need to adequately assess the growing epidemiologic literature between prenatal BPA exposure and children’s behavior for decision-making. This implies the active participation of experienced epidemiologists in future BPA risk assessments. When both the context and data are taken together, the in vitro, in vivo rodent, some non-human primate studies and a growing epidemiologic evidence from birth cohorts converge into the same direction: BPA is a probable developmental neurotoxicant at low doses [2–4, 7]. Radical measures do not appear realistic or the best solution. But inaction at this point may be irresponsible. Accordingly, the precautionary principle must be followed and stringent preventive policies, such as the banning of BPA in food contact materials and thermal receipts, should be progressively implemented worldwide with a focus on the utilization of safer substitutes.

## Supplementary information

**Additional file 1: Supplemental Table 1.** Summary of results reported in mother-child cohorts addressing the relationship between prenatal urinary BPA concentrations and children’s behavior. **Supplemental Table 2.** Comparison of prenatal vs. postnatal associations between urinary BPA concentrations and children’s behavior. **Supplemental Table 3.** Comparison of associations among the cohorts that assessed both cognitive and other behavioral outcomes in relation to prenatal and/or postnatal BPA exposure.
