# Supplementary material for: Bisphenol A shapes children’s brain and behavior: towards an integrated neurotoxicity assessment including human data
Source: Environ Health. 2020 Jun 9;19:66. doi: 10.1186/s12940-020-00620-y (PMC7285610; doi:10.1186/s12940-020-00620-y)
Supplement: Supplementary file 1 — Additional file 1: Supplemental Table 1. Summary of results reported in mother-child cohorts addressing the relationship between prenatal urinary BPA concentrations and children’s behavior. Supplemental Table 2. Comparison of prenatal vs. postnatal associations between urinary BPA concentrations and children’s behavior. Supplemental Table 3. Comparison of associations among the cohorts that assessed both cognitive and other behavioral outcomes in relation to prenatal and/or postnatal BPA exposure. [file 12940_2020_620_MOESM1_ESM.docx]

**Supplementary Appendix**

**Bisphenol A shapes children’s brain and behavior: Towards an integrated neurotoxicity assessment including human data**

Vicente Mustieles & Mariana F. Fernández

**Supplemental Table 1.** Summary of results reported in mother-child cohorts addressing the relationship between prenatal urinary BPA concentrations and children’s behavior.

**Supplemental Table 2.** Comparison of prenatal *vs.* postnatal associations between urinary BPA concentrations and children’s behavior.

**Supplemental Table 3.** Comparison of associations among the cohorts that assessed both cognitive and other behavioral outcomes in relation to prenatal and/or postnatal BPA exposure.

**References.**

**Supplemental Table 1.** Summary of results reported in mother-child cohorts addressing the relationship between prenatal urinary BPA concentrations and children’s behavior.

| **Cohort study** | **Sample size** | **Prenatal urine(s) samples** | **Association*** | **Internalizing or externalizing** | **Boys, girls or both** |
| --- | --- | --- | --- | --- | --- |
| **HOME (USA)**  Braun et al. 2009 [1] | 249 | 3 (gestation weeks 16^th^ and 26^th^, and delivery) | Yes | Externalizing at 2 yrs. | Girls |
| Braun et al., 2011 [2] | 244 |  | Yes | Internalizing at 3 yrs. |  |
| Braun et al., 2014 [3] | 175 | 2 (gestation weeks 16^th^ and 26^th^) | No | Autistic behaviors at 4-5 yrs. | n/a |
| Braun et al., 2017 [4] | 178 | 2 (gestation weeks 16^th^ and 26^th^) | Yes | Externalizing behavior trajectory throughout ages 2, 3, 4, 5 and 8 | Girls |
| **MSCEHS (USA)**  Miodovnik et al., 2011 [5] | 137 | 1 (gestation week 31^th^) | No  (Yes, when 6 outliers were removed from the analysis) | Autistic behaviors at 7-9 yrs. | n/a |
| **CCCEH (USA)**  Perera et al. 2012 [6] | 198 | 1 (gestation week 34^th^) | Yes | Both at 3-5 yrs. | Boys |
| Roen et al., 2015 [7] | 250 |  | Yes | Both at 7-9 yrs. |  |
| Perera et al., 2016 [8] | 241 |  | Yes | Internalizing at 10-12 yrs. |  |
| **CHAMACOS (USA)**  Harley et al. 2013 [9] | 292 | 2 (gestation weeks 14^th^ and 26^th^) | Yes | Mostly internalizing, but also aggressive behavior at 7 yrs. | Boys |
| **SFFII (USA)**  Evans et al., 2014 [10] | 153 | 1 (gestation week 27^th^) | Yes | Both at 5 yrs. | Boys |
| **INMA-Sabadell (Spain)**  Casas et al., 2015 [11] | 438 | 2 (1^st^ and 3^rd^ trimester) | Yes | Externalizing at 7 yrs. | Boys |
| **EDEN (France)**  Philippat et al., 2017 [12] | 529 | 1 (2^nd^ trimester) | Yes | Internalizing at 3 yrs. Externalizing at 5 yrs. | Only boys |
| **MIREC (Canada)**  Braun et al., 2017 [13] | 812 | 1 (gestation week 12^th^) | Yes | Internalizing at 3 yrs. | Boys |
| **EDC (Korea)**  Lim et al., 2017 [14] | 304 | 1 (gestation week 20^th^) | Yes | Externalizing (Social problems) at 4 yrs. | Girls |
| **OCC (Denmark)**  Jensen et al., 2019 [15] | 658 | 1 (gestation week 28^th^) | No | At 2-4 yrs. | n/a |
| **CHECK (Korea)**  Kim et al., 2018 [16] | 140 | 1 (delivery) | No | At 1-2 yrs. | n/a |
| **APrON (Canada)**  Grohs et al., 2019 [17] | 98 | 1 (gestation week 17^th^) | Yes | Internalizing at 4-5 years | Both |
| **S-MBCS (China)**  Li et al., 2020 [18] | 745 | 1 (gestation weeks 12^th^-16^th^) | Yes | Both at 2-4 yrs. | Boys |

**A p-value ≤ 0.05 defined the existence of an association. However, other parameters such as the internal validity of associations were also considered. n/a: Not applicable, since no association was reported. APrON (Alberta Pregnancy Outcomes and Nutrition); CHAMACOS (Center for the Health Assessment of Mothers and Children of Salinas); CHECK (Children's Health and Environmental Chemicals in Korea); CCCEH (Columbia Center for Children’s Environmental Health); EDC (Environment and Development of Children); EDEN (Study on the Pre- and Early Postnatal Determinants of Child Health and Development); HOME (Health Outcomes and Measures of the Environment Study); INMA (Environment and Childhood Project); MIREC (Maternal-Infant Research on Environmental Chemicals); MSCEHS (Mount Sinai Children’s Environmental Health Study); OCC (Odense Child Cohort); S-MBS (Shanghai-Minhang Birth Cohort Study); SFFII (Study for Future Families II).*

**Supplemental Table 2.** Comparison of prenatal vs. postnatal associations between urinary BPA concentrations and children’s behavior.

| **Cohort** | **Sample size** | **Prenatal urine(s) samples** | **Postnatal urine(s) samples** | **Prenatal association*** | **Postnatal association*** |
| --- | --- | --- | --- | --- | --- |
| **HOME (USA)**  Braun et al. 2011 [2] | 244 | 3 (gestation weeks 16^th^ and 26^th^, and delivery) | Average of 3 urines (at 1, 2 and 3 yrs.) | Yes  (at 3 yrs.) | No |
| Stacy et al., 2017 [19] | 228 | 2 (gestation week 26^th^ and delivery) | 6 (1, 2, 3, 4, 5 and 8 yrs.) | Yes | Yes  (only at 8 yrs.) |
| **CCCEH (USA)**  Perera et al. 2012 [6] | 198 | 1 (gestation week 34^th^) | 2 (at 3 and 5 yrs.) | Yes  (at 3-5 yrs.) | No  (at 3-5 yrs.) |
| Roen et al., 2015 [7] | 250 |  | 2 (at 3 and 5 yrs.) | Yes  (at 7-9 yrs.) | Yes  (at 7-9 yrs.) |
| Perera et al., 2016 [8] | 241 |  | 2 (at 3 and 5 yrs.) | Yes  (at 10-12 yrs.) | No  (at 10-12 yrs.) |
| **CHAMACOS (USA)**  Harley et al. 2013 [9] | 292 | 2 (gestation week 14^th^ and 26^th^) | 1 (at 5 yrs.) | Yes  (at 7 yrs.) | Yes  (at 7 yrs.) |
| **EDC (Korea)**  Lim et al., 2017 [14] | 304 | 1 (gestation week 20^th^) | 1 (at 4 yrs.) | Yes  (at 4 yrs.) | Yes  (at 4 yrs.) |
| **APrON (Canada)**  Grohs et al., 2019 [17] | 98 | 1 (gestation week 17^th^) | 1 (at 3-4 yrs.) | Yes  (at 2-5 yrs.) | No  (at 3-5 yrs.) |

**A p-value ≤ 0.05 defined the existence of an association. However, other parameters such as the internal validity of associations were also considered. APrON (Alberta Pregnancy Outcomes and Nutrition); CHAMACOS (Center for the Health Assessment of Mothers and Children of Salinas); CCCEH (Columbia Center for Children’s Environmental Health); EDC (Environment and Development of Children); HOME (Health Outcomes and Measures of the Environment Study).*

**Supplemental Table 3.** Comparison of associations among the cohorts that assessed both cognitive and other behavioral domains in relation to prenatal and/or postnatal BPA exposure.

| **Cohort** | **Sample size** | **Prenatal/postnatal urine sample(s)** | **Associations with externalizing/internalizing behaviors*** | **Associations with cognition*** |
| --- | --- | --- | --- | --- |
| **HOME**  Braun et al., 2011 [2] | 244 | 3 (gestation week 16^th^ and 26^th^, and birth) | Several | Any |
| Stacy et al., 2017 [19] | 228 | 6 (1, 2, 3, 4, 5 and 8 yrs.) | Several | Some  (Only cross-sectional at 8 yrs.) |
| **INMA-Sabadell**  Casas et al., 2015 [11] | 438 | 2 (1^st^ and 3^rd^ trimester) | Some | Any |
| **EDEN**  Philippat et al., 2017 [12]  Nakiwala et al., 2019 [20] | 546 | 1 (2^nd^ trimester) | Several | Any |
| **MIREC**  Braun et al., 2017 [13] | 812 | 1 (gestation week 12^th^) | Lots | Any, except poorer working memory in boys |
| **OCC**  Jensen et al., 2019 [15] | 658 | 1 (gestation week 28^th^) | Any | Poorer language development in boys at 2-3 yrs. |
| **INMA-Granada**  Pérez-Lobato et al., 2016 [21]  Rodríguez-Carrillo et al., 2019 [22] | 300 | 1 (at 9-11 yrs.) | Several | Any, except poorer working memory at 9-11 yrs. (only boys) |
| **NECAT**  Masejerian et al., 2012a [23] and 2012b [24] | 434 | BPA exposure estimated based on number of composite dental restorations | Several | Any |

**A p-value ≤ 0.05 defined the existence of an association. However, other parameters such as the internal validity of associations were also considered. n/a: Not applicable, since no association was reported. EDEN (Study on the Pre- and Early Postnatal Determinants of Child Health and Development); HOME (Health Outcomes and Measures of the Environment Study); INMA (Environment and Childhood Project); MIREC (Maternal-Infant Research on Environmental Chemicals); OCC (Odense Child Cohort); NECAT (New England Children’s Amalgam Trial).*

**References:**

1. Braun JM, Yolton K, Dietrich KN, Hornung R, Ye X, Calafat AM, et al. Prenatal bisphenol A exposure and early childhood behavior. Environ Health Perspect. 2009; 117:1945–52.

2. Braun JM, Kalkbrenner AE, Calafat AM, Yolton K, Ye X, Dietrich KN, et al. Impact of early-life bisphenol A exposure on behavior and executive function in children. Pediatrics. 2011; 128:873–82.

3. Braun JM, Kalkbrenner AE, Just AC, Yolton K, Calafat AM, Sjödin A, et al. Gestational exposure to endocrine-disrupting chemicals and reciprocal social, repetitive, and stereotypic behaviors in 4- and 5-year-old children: The HOME study. Environ Health Perspect. 2014; 122:513–20.

4. Braun JM, Yolton K, Stacy SL, Erar B, Papandonatos GD, Bellinger DC, et al. Prenatal environmental chemical exposures and longitudinal patterns of child neurobehavior. Neurotoxicology. 2017; 62:192–9.

5. Miodovnik A, Engel SM, Zhu C, Ye X, Soorya L V, Silva MJ, et al. Endocrine disruptors and childhood social impairment. Neurotoxicology. 2011; 32:261–7.

6. Perera F, Vishnevetsky J, Herbstman JB, Calafat AM, Xiong W, Rauh V, et al. Prenatal bisphenol a exposure and child behavior in an inner-city cohort. Environ Health Perspect. 2012; 120:1190–4.

7. Roen EL, Wang Y, Calafat AM, Wang S, Margolis A, Herbstman J, et al. Bisphenol A exposure and behavioral problems among inner city children at 7-9 years of age. Environ Res. 2015; 142:739–45.

8. Perera F, Nolte ELR, Wang Y, Margolis AE, Calafat AM, Wang S, et al. Bisphenol A exposure and symptoms of anxiety and depression among inner city children at 10–12 years of age. Environ Res. 2016; 151:195–202.

9. Harley KG, Aguilar Schall R, Chevrier J, Tyler K, Aguirre H, Bradman A, et al. Prenatal and postnatal bisphenol A exposure and body mass index in childhood in the CHAMACOS cohort. Environ Health Perspect. 2013; 121:514–20.

10. Evans SF, Kobrosly RW, Barrett ES, Thurston SW, Calafat AM, Weiss B, et al. Prenatal bisphenol A exposure and maternally reported behavior in boys and girls. Neurotoxicology. 2014; 45:91–9.

11. Casas M, Forns J, Martínez D, Avella-García C, Valvi D, Ballesteros-Gómez A, et al. Exposure to bisphenol A during pregnancy and child neuropsychological development in the INMA-Sabadell cohort. Environ Res. 2015; 142:671–9.

12. Philippat C, Nakiwala D, Calafat AM, Botton J, De Agostini M, Heude B, et al. Prenatal exposure to nonpersistent endocrine disruptors and behavior in boys at 3 and 5 years. Environ Health Perspect. 2017; 125(9):097014.

13. Braun JM, Muckle G, Arbuckle T, Bouchard MF, Fraser WD, Ouellet E, et al. Associations of prenatal urinary bisphenol A concentrations with child behaviors and cognitive abilities. Environ Health Perspect. 2017; 125(6):067008.

14. Lim Y-H, Bae S, Kim B-N, Shin CH, Lee YA, Kim JI, et al. Prenatal and postnatal bisphenol A exposure and social impairment in 4-year-old children. Environ Health. 2017; 16(1):79.

15. Jensen TK, Mustieles V, Bleses D, Frederiksen H, Trecca F, Schoeters G, et al. Prenatal bisphenol A exposure is associated with language development but not with ADHD-related behavior in toddlers from the Odense Child Cohort. Environ Res. 2019; 170:398–405.

16. Kim S, Eom S, Kim H-J, Lee JJ, Choi G, Choi S, et al. Association between maternal exposure to major phthalates, heavy metals, and persistent organic pollutants, and the neurodevelopmental performances of their children at 1 to 2years of age- CHECK cohort study. Sci Total Environ. 2018; 624:377–84.

17. Grohs MN, Reynolds JE, Liu J, Martin JW, Pollock T, Lebel C, et al. Prenatal maternal and childhood bisphenol a exposure and brain structure and behavior of young children. Environ Health. 2019; 18(1):85.

18. Li F, Yang F, Li D-K, Tian Y, Miao M, Zhang Y, et al. Prenatal bisphenol A exposure, fetal thyroid hormones and neurobehavioral development in children at 2 and 4 years: A prospective cohort study. Sci Total Environ. 2020; 722:137887.

19. Stacy SL, Papandonatos GD, Calafat AM, Chen A, Yolton K, Lanphear BP, et al. Early life bisphenol A exposure and neurobehavior at 8 years of age: Identifying windows of heightened vulnerability. Environ Int. 2017; 107:258–65.

20. Nakiwala D, Peyre H, Heude B, Bernard JY, Béranger R, Slama R, et al. In-utero exposure to phenols and phthalates and the intelligence quotient of boys at 5 years. Environ Health. 2018; 17(1):17.

21. Perez-Lobato R, Mustieles V, Calvente I, Jimenez-Diaz I, Ramos R, Caballero-Casero N, et al. Exposure to bisphenol A and behavior in school-age children. Neurotoxicology. 2016; 53:12–9.

22. Rodríguez-Carrillo A, Mustieles V, Pérez-Lobato R, Molina-Molina JM, Reina-Pérez I, Vela-Soria F, et al. Bisphenol A and cognitive function in school-age boys: Is BPA predominantly related to behavior? Neurotoxicology. 2019; 74:162–71.

23. Maserejian NN, Trachtenberg FL, Hauser R, McKinlay S, Shrader P, Tavares M, et al. Dental composite restorations and psychosocial function in children. Pediatrics. 2012; 130:e328-38.

24. Maserejian NN, Trachtenberg FL, Hauser R, McKinlay S, Shrader P, Bellinger DC. Dental composite restorations and neuropsychological development in children: Treatment level analysis from a randomized clinical trial. Neurotoxicology. 2012; 33:1291–7.
